# Translating the Immunobiology of SBRT to Novel Therapeutic Combinations for Advanced Prostate Cancer

**DOI:** 10.3389/fonc.2020.00830

**Published:** 2020-05-27

**Authors:** Victor R. Adorno Febles, Seth Blacksburg, Jonathan A. Haas, David R. Wise

**Affiliations:** ^1^Perlmutter Cancer Center, Langone Medical Center, New York University, New York, NY, United States; ^2^New York University Winthrop Hospital, Mineola, NY, United States

**Keywords:** SBRT, prostate cancer, immunotherapy, ADT, minimal residual disease, androgen suppression therapy

## Abstract

Stereotactic body radiotherapy (SBRT) is an increasingly used radiation modality for the treatment of both localized and metastatic prostate cancer. Substantial data suggests that prostate cancer may be more sensitive to higher doses of radiation per fraction due to its low α/β ratio. This increased sensitivity raises important questions as to how SBRT should be combined with systemic therapy for clinically significant prostate cancer, including whether androgen deprivation therapy retains its beneficial effects when combined with SBRT. Furthermore, pre-clinical and clinical data suggest pronounced immunomodulatory effects of SBRT, including observed improvements in T cell priming and trafficking. These data support investigational strategies combining SBRT with immunotherapy. Here we aim to review the data for the use of SBRT in both the local and metastatic disease settings as well as ongoing translational and clinical research examining combinations with ADT, immunotherapy and other targeted agents.

## The Shift to Hypofractionated Radiation Therapy in Prostate Cancer

Prostate cancer is the second most common cancer diagnosis in men (1). Seventy seven percent of the men diagnosed will present with localized disease (2). For these men, treatment options include active surveillance, surgery or radiation with or without androgen deprivation therapy (ADT) (3). Choice of therapy is influenced by risk stratification based on prostate specific antigen (PSA), clinical stage, Gleason grade group, disease burden on biopsy, PSA density and imaging (4). For men electing radiation, conventional radiotherapy consisting of 40 to 45 treatments over 8 to 9 weeks was the previous standard. Multiple trials have shown that 4 to 6 weeks of moderately hypofractionated RT is not inferior to conventional fractionation with respect to oncologic outcomes. However, data beyond 5 years are not yet available (5–8). A large meta-analysis confirmed similar therapeutic outcomes, albeit with a small increase in acute gastrointestinal (GI) toxicity in patients receiving moderate hypofractionation (9). However, a more recent report on quality of life (QOL) assessment for patients receiving hypofractionated vs. conventional radiotherapy as part of a phase 3 trial showed no difference in any of the QOL domains between treatment arms (10). Based on the results of these prospective studies, a 2018 consensus expert panel recommended moderate hypofractionation as a standard option for patients presenting with localized disease who are to be treated with EBRT (11). Other than the advantages of shorter treatment schedules, some reports have also shown a health care cost benefit to the moderately hypofractionated schedules when compared to the cost of conventional fractionation (12). With improvements in technology, further research has evaluated even shorter courses of radiation for both localized and metastatic disease. Stereotactic body radiotherapy is a form of external beam radiotherapy that administers higher doses of radiation per fraction of therapy. This allows for high doses of radiation to be administered in a short time; usually 5 days of treatment or less.

## Radiobiology Of SBRT In Prostate Cancer

Traditional External Beam Radio Therapy (EBRT) for prostate cancer is delivered in fractions of 1.8 to 2.0 Gy over 8 to 9 weeks. This fractionation leads to high doses of radiation to the tumor while allowing time for normal tissues to recover via sublethal damage repair. However, significant data suggests that prostate cancer may be more sensitive to higher doses of radiation per fraction due to its low α / β ratio. The α / β ratio or repair capacity, describes the tumor response to changes in fractionation. A high α / β ratio reflects a small sensitivity to changes in fractionation whereas a low value reflects a large sensitivity to changes in fractionation (13). An initial report estimated prostate cancer α/β value as 1.5 Gy, which supports hypofractionation (13). A more recent meta-analysis of 8 fractionation trials including 7,946 patients estimated an α/β ratio of 1.2 Gy consistent with previous findings (14). The combination of a low α/ β ratio with the potential for better control with higher doses supports shorter fractionation treatment courses.

## SBRT For Localized Disease

Multiple retrospective and prospective trials have evaluated the role of SBRT in patients with localized prostate cancer (see Table 1). SHARP was the first prospective clinical trial evaluating 33.5 Gy in 5 fractions. In this study of 40 patients with a median follow up of 41 months, SBRT achieved a 90% rate of biochemical progression free survival (bPFS) at 48 months and no late grade 3 or higher toxicity (15). Multiple other trials have shown similar toxicity and efficacy to conventional radiation. In a recently published systematic review and meta-analysis including over 6,000 patients with a median follow up of 39 months across all patients, SBRT led to 5 and 7 year bRFS rates of 95.3 and 93.7%, respectively (16). By two years after SBRT, both urinary and bowel toxicity returned to baseline. Of note, there was a significant association between increasing dose of SBRT and improved biochemical control at the cost of worse late grade 3 or higher urinary toxicity. ADT was used in only 15% of the patients, which precludes meaningful analysis of its impact on outcomes. As such, the role of ADT in the context of SBRT is evolving.

**Table 1:** Notable SBRT trials in prostate cancer.

**Title**	**Setting**	**Intervention/Treatment**	**Phase**
Stereotactic hypofractionated accurate radiotherapy of the prostate (SHARP), 33.5 Gy in five fractions for localized disease: first clinical trial results.	Localized disease	Non-Randomized: 33.5 Gy in 5 Fractions	Phase 1/II Trial
Ultra-hypofractionated vs. conventionally fractionated radiotherapy for prostate cancer: 5-year outcomes of the HYPO-RT-PC randomized, non-inferiority, phase 3 trial.	Localized disease	Randomized: 42.7 Gy in 7 Fractions 3 days per week for 2.5 weeks vs. 78 Gy in 39 Fractions, 5 days per week for 8 weeks.	Phase III Trial
Intensity-modulated fractionated radiotherapy vs. stereotactic body radiotherapy for prostate cancer (PACE-B): acute toxicity findings from an international, randomised, open-label, phase 3, non-inferiority trial.	Localized disease	Randomized: 36.25 Gy in 5 Fractions over 1-2 weeks. vs. 78 Gy in 39 Fractions over 7-8 weeks or 62 Gy in 20 fractions over 4 weeks.	Phase III Trial
Radiotherapy to the primary tumour for newly diagnosed, metastatic prostate cancer (STAMPEDE): a randomised controlled phase 3 trial.	Metastatic disease	Randomized: Standard of care vs. Standard of care + radiotherapy to primary tumor. RT consisted of either 55 Gy in 20 Fractions over 4 weeks or 36 Gy in 6 Fractions over 6 weeks.	Phase III Trial
Stereotactic ablative radiotherapy vs. standard of care palliative treatment in patients with oligometastatic cancers (SABR-COMET): a randomised, phase 2, open-label trial.	Metastatic disease (Prostate as well as other solid tumor types)	Randomized: standard of care vs. Standard of care + SBRT to up to 5 metastatic lesions. Dose ranged from 8 Gy in one Fraction to 30 Gy in 10 Fractions.	Phase II Trial
Surveillance or Metastasis-Directed Therapy for Oligometastatic Prostate Cancer Recurrence: A Prospective, Randomized, Multicenter Phase II Trial. (STOMP)	Metastatic disease	Randomized: surveillance vs. MDT with metastasectomy or SBRT to up to 3 metastatic lesions. Radiation consisted of 30 Gy in 3 Fractions.	Phase II Trial
Outcomes of Observation vs. Stereotactic Ablative Radiation for Oligometastatic Prostate Cancer: The ORIOLE Phase 2 Randomized Clinical Trial.	Metastatic disease	Randomized: observation vs. SBRT to up to 3 metastatic lesions. Doses ranged from 19.5 to 48 Gy in 3 to 5 Fractions.	Phase II Trial
Stereotactic Abative Body Radiotherapy (SABR) for Oligometastatic Prostate Cancer: A Prospective Clinical Trial. (POPSTAR)	Metastatic disease	Non-Randomized 20 Gy in 1 Fraction to up 3 metastatic lesions.	Phase II Trial

Of these trials, HYPO-RT-PC was the first randomized trial comparing ultra-hypofractionated radiation with conventionally fractionated radiation in men with intermediate to high risk prostate cancer (17). This phase 3, non-inferiority trial randomized patients to receive 42.7 Gy in seven fractions or 78 Gy in 39 fractions; no ADT was allowed. In total, 1,200 patients were randomized into both treatment arms with most participants consisting of patients with intermediate risk disease and only 11% with high risk disease. Ultra-hypofractionation was found to be non-inferior to conventional fractionation. Failure free survival at 5 years was 84% in both groups. The ultra-hypofractionated schedule was weakly associated with worse urinary toxicity at the end of radiotherapy compared to standard fractionation, 28 vs. 23%, respectively (*P* = 0.057). There were no significant differences in grade 2 or worse urinary or bowel late toxicity between the two groups with the exception of increased urinary toxicity in the hypofractionated group at 1 year (6 vs. 2 %) in the conventional fractionation group (*P* = 0.0037). In the PACE-B trial, 874 men were randomly assigned to conventionally fractionated (78 Gy in 39 fractions) or moderately hypofractionated radiotherapy (62 Gy in 20 fractions) vs. SBRT (36.25 Gy in 5 fractions) (18). Grade 2 or higher GI or GU toxicity did not differ between the groups. In this initial acute toxicity report, there was no evidence that shortening radiotherapy courses increased acute toxicity, which contrasted with the increased acute toxicity observed in HYPO-RT-PC trial. The authors propose that the higher toxicity observed in HYPO-RT-PC might be secondary to differences in toxicity assessment, radiation dose or conformal radiotherapy technique (18).

Based on the above evidence, SBRT can now be considered as a reasonable alternative to conventionally fractionated regimens and is included as a treatment option for patients with low and intermediate risk disease in recently updated ASTRO/ASCO/AUA guidelines (19). Of note, while prospective trials have used different SBRT treatment schedules, delivery of SBRT on consecutive days is not recommended given the reported increased risk of late GI and GU toxicity with this schedule (19). In addition, PATRIOT, a phase II trial which randomized 152 patients to receive 5 fractions of SBRT for a total of 40 Gy in either a weekly or every other day schedule reported less acute GI and GU toxicity with the weekly schedule (20). With these new guidelines a shift in the treatment of patients with localized disease has been noted. A recent study showed that the proportion of patients receiving SBRT increased from 0.9% in 2004 to 19.5% in 2015. Moderate hypofractionation also increased in use but to a lesser extent with 2.7% receiving moderate hypofractionation in 2004 and 4.7% in 2015. On the other hand, the use of conventional fractionation dramatically decreased (14,699 patients treated with conventional fractionation in 2009 decreasing to 1,492 in 2011) (21). In this report, the most used fractionation schedule was 36.25 Gy in 5 fractions. While preliminary data have demonstrated promising outcomes for patients treated with high risk disease, the use of SBRT remains non-standard for this patient population and should be utilized in the setting of a clinical trial (22).

## SBRT For Metastatic Prostate Cancer

Metastatic prostate cancer remains incurable and accounts for over 30,000 deaths in the United States (US) each year (2). ADT is the backbone of therapy for patients with metastatic disease. Multiple recent clinical trials have demonstrated life-prolonging benefits of abiraterone acetate, enzalutamide, apalutamide, and docetaxel when added to ADT for patients with hormone-sensitive and castration-resistant metastatic prostate cancer (23–25). The toxicities of ADT and novel hormonal agents—decreased bone density, increased risk of cardiovascular events, sexual dysfunction and neuropsychiatric symptoms—support the need for integrating metastasis-directed therapies into the treatment paradigm (26, 27). The success of SBRT in palliating painful metastatic bone lesions and its favorable toxicity profile led to increasing interest in SBRT for patients with limited metastatic disease with the goal of either sparing patients the toxicity from systemic therapies or augmenting the effects of existing systemic therapy without leading to undue toxicity (see Table 1).

Support for the latter paradigm came from the phase 3 STAMPEDE study, which demonstrated improved OS in patients with low-volume metastatic disease receiving radiation to the primary prostate tumor (28). In this trial, 2061 patients were randomly allocated to either standard of care (SOC) or SOC plus radiotherapy (55 Gy in 20 fractions or 36 Gy in 6 fractions). With a median follow up of 37 months, there was no survival advantage to radiotherapy in the general cohort. The study stratified patients by metastatic burden as defined in CHAARTED (29), in which high metastatic burden was defined as the presence of visceral metastasis or four or more bone metastases with one or more outside the vertebral bodies or pelvis. STAMPEDE demonstrated an improved OS in patients with low metastatic burden at baseline who were allocated to radiotherapy to the primary tumor. (HR 0.68, *p* = 0.007) (28). This data provided further support for radiation therapy as a means to prolong OS in patients with low volume metastatic prostate cancer.

Whether this approach can be improved upon through RT to all sites of disease as opposed to the primary tumor alone in patients with oligometastatic disease is currently under investigation. The oligometastatic paradigm suggests that in some patients with low volume metastatic disease, the disease is not completely disseminated, and thus ablative therapies could provide durable disease control (30). SABR-COMET was a recently reported randomized phase 2 trial of stereotactic ablative radiotherapy vs. standard palliative treatment in patients with oligometastatic cancers (31). In this study, patients with a controlled primary tumor and one to five metastatic lesions were randomized to either palliative SOC treatment alone or SOC plus SBRT to all metastatic lesions. A total of 99 patients were randomized and with a median follow up of 25 months in the control group vs. 26 months in the SABR group, the median OS reported was 28 months in the control group vs. 41 months in the SABR group. However, grade 2 or worse adverse events occurred in 9% of controls vs. 29% of those in the SABR group (*P* = 0.026). Treatment related deaths occurred in 3 patients in the SABR group compared to none in the control group. In this prospective study, SABR was associated with a 13-month improvement in median OS and a doubling of median PFS at the cost of an increase in toxicity and a 4.5% risk of treatment related mortality (31). Of note, out of the 99 patients included in this trial only 16 had prostate cancer, 14 of these were randomized to the radiation arm and the other 2 to the control arm. Given the limited number of prostate cancer patients in this study, a disease specific benefit cannot be proven. However, the results are encouraging for using SBRT in addition to SOC therapy in a disease agnostic manner.

The first prospective clinical trial examining the utility of metastasis directed therapy (MDT) in prostate cancer was STOMP. This was a multicenter phase 2 trial which included asymptomatic prostate cancer patients presenting with up to 3 extracranial metastatic lesions on choline PET-CT after curative intent therapy to the primary. Patients were randomized to either surveillance or MDT with either metastatectomy or SBRT to all lesions. An initial report revealed that at median follow up of 3 years ADT free survival was 13 months in the surveillance group vs. 21 months in the MDT group (32). An update of this trial including five year results was recently presented in abstract form at GU ASCO 2020 and demonstrated that 5 year ADT survival was 8% for the surveillance group vs. 34% for MDT group (HR 0.57 [80% CI: 0.38−0.84]). In addition, 5-year CRPC free survival, a secondary endpoint of the study, was 53% for the surveillance group and 76% for the MDT group (HR 0.62 [80% CI 0.35–1.09]) (33). These results further confirmed the significant difference in ADT-free survival for those patients receiving MDT.

POPSTAR was a single arm prospective trial which evaluated the feasibility and tolerability of a single fraction of stereotactic ablative radiotherapy to metastatic sites in men with prostate cancer and up to 3 metastatic lesions on sodium fluoride PET CT. A total of 33 consecutive patients received stereotactic ablative RT to a total of 50 oligometastases. Local PFS at 2 years was 97%, while distant PFS was 39%. For patients not on ADT, the 2-year freedom from ADT was 48% (34).

Lastly, ORIOLE, was a phase 2 randomized study in which patients with hormone sensitive prostate cancer and 1 to 3 asymptomatic metastases detected by conventional imaging who had not yet received ADT were randomized to SABR or observation. 54 men were randomized into the treatment arms. Progression at 6 months was observed in 19% of those receiving SABR vs. 61% of those undergoing observation (*P* = 0.005). No toxic effects of grade 3 or greater were observed. In addition, peripheral blood mononuclear cells (PBMC) were collected at baseline and at 90 days for deep sequencing of T-cell receptor DNA. Interestingly, differential clonotype abundance appeared more pronounced in SABR arm with significantly expanded clones when compared to observation arm (35).

Given the current body of evidence, the Italian association of radiotherapy and clinical oncology (AIRO) released a consensus statement that supported the use of SBRT as an alternative to ADT to delay systemic recurrence in patients with oligometastatic disease and up to 3 lymph node or bone metastasis (36). The use of SBRT to asymptomatic metastatic sites remains non-standard, however data from SABR-COMET, POPSTAR, ORIOLE and additional trials underway points to this as a promising area of investigation. These studies provide evidence that radiation therapy can alter the natural history of a subset of metastatic prostate cancers. Whether this mechanism proceeds through tumor debulking, direct synergy with AR-targeted therapies, or immunomodulation remains unknown. These clinical data coupled with an increasing understanding of the immunobiological effects of radiation therapy has spurred innovative clinical trials designed to address these questions.

## SBRT and Tumor Immunology

A body of preclinical and clinical data suggest that radiation therapy promotes T cell priming through the release of tumor antigens and pro-inflammatory cytokines (37). Both innate and adaptive mechanisms have been highlighted as critical for the effects of high dose radiation on T cell priming. Innate effects include the positive effect on dendritic cell-mediated cross-presentation to CD8+ T cells in a type 1 interferon-dependent fashion (38). Ionizing radiation has also been shown to promote improved T cell trafficking to the tumor through upregulation of critical chemokines (39). Indeed, when combining immunotherapy and radiotherapy, an abscopal treatment response was observed in a mouse model of castrate resistant prostate cancer (40).

However, the dose and fractionation schedules required to achieve this immunologic priming are unclear (41). Several studies have suggested that ablative or hypofractionated radiation is associated with increased activation of antitumor CD8+ T cells when compared to conventional fractionation (42–44). For instance, a report compared the effects of a single dose of 15 Gy to a regimen of 5 fractions of 3 Gy on a murine model of melanoma and found increased numbers of IFN-γ secreting cells and increased cell lysis by CD8+ T-cells when compared to the fractionated scheme (43). Another study reported tumor rejection after a single 20 Gy dose of ablative RT in an immunocompetent murine model of B16 melanoma. Interestingly, this effect was not seen in immunodeficient mice or mice that had been depleted of CD8+ T cells (42). In this same report, immunocompetent mice treated with a fractionated scheme of 4 x 5 Gy showed a similar tumor relapse rate to that of T cell-depleted mice, suggesting that fractionated regimens may inadequately prime or mobilize tumor infiltrating lymphocytes (41). In further support of hypofractionated schemes, high dose radiation was found to reverse the immunosuppressive tumor microenvironment of CT26 and MC38 colon tumors by leading to increased CD8+ T cell infiltrate and reduced myeloid derived suppressor cells. These changes were found to be dependent on antigen presenting dendritic cells and secretion of IFN-γ. This effect was not seen with extended fractionated radiation regimens (45).

Whether SBRT-induced DNA damage leads to increased expression of neoantigens and an expanded T cell repertoire remains an open question. A recent study tested conventional fractionation (5 x 2 Gy) in combination with an anti-PD-1 antibody and reported local and systemic immune responses in mice with CT26 colon carcinoma xenografts (46). In this model, there was a high level of concordance between T-cell receptors from irradiated and non-irradiated tumors, whereas only 0.5% of T cells were unique to the irradiated tumor. This suggests that most of the reactive T cells in this model were responding to pre-existing antigens rather than neo-antigens created by radiation-induced injury. Therefore, in some circumstances, radiation may lead to increased release of tumor associated antigens (TAA) rather than directly generating new TAA. Indeed, many studies have reported enhanced priming of T cell responses after treatment with hypofractionated RT due to increased presentation of TAA to CD8+ T cells in draining lymph nodes (42, 43, 47, 48). Whether SBRT doses in certain circumstances can generate an increased number of TAAs remains an area of inquiry.

Whether these immunomodulatory effects are related to the fractionation scheme or the biological dose achieved with ablative therapy was further tested in a study with syngeneic mouse lung and melanoma tumors under the same biologically equivalent dose (BED) (49). In this study, when compared with conventional fractionation, ablative therapy suppressed recruitment of MDSCs, which in turn allowed for increased cytotoxic activity of CD8+ T cells. Treating with anti-PD-L1 antibody further improved the effects of ablative therapy on mouse survival (49). In further support of this strategy, PD-1 blockade can reverse adaptive immune resistance when combined with high dose hypofractionated therapy but not with daily fractionation (50). Lastly, in addition to its effect on the tumor immune microenvironment, other studies have shown that hypofractionated RT can alter the tumor stroma (41). For instance, a recent study developed a dynamic model that confirmed indirect tumor cell death caused by SBRT through its effect on tumor-associated endothelial cell vascular damage (51).

In addition to the above pre-clinical data a recent phase 1 clinical trial also suggested a beneficial immune effect to SBRT in combination with immunotherapy (52). In this study, patients with advanced solid tumors progressing on standard therapy received SBRT to up to four metastatic lesions followed by pembrolizumab. Pre and post SBRT biopsy specimens were analyzed in a subset of patients. In the 68 patients with imaging follow up, the objective response rate was 13.2%. Expression of interferon gamma-associated genes from post-SBRT tumor biopsy specimens significantly correlated with non-irradiated tumor response further suggesting a complementary role of SBRT in combination with immunotherapy.

Another report demonstrated that SBRT and CTLA-4 blockade with ipilimumab induced systemic anti-tumor T cells in chemo-refractory metastatic non-small cell lung cancer, a clinical context in which anti-CTLA-4 antibodies had previously failed to demonstrate efficacy alone or in combination with chemotherapy (53). In this study, increased serum interferon β after radiation and early dynamic changes in blood T cell clones were the strongest predictors of response. Whether radiotherapy can reliably induce immune responses in combination with immune checkpoint inhibition remains an area of active investigation (54). Similar prospective trials are currently addressing the potential impact of SBRT on altering the natural history of prostate cancer and will provide a rich source of correlative markers to address the effects on the TME.

## Translating The Biology of SBRT To The Clinic

SBRT is an increasingly used treatment modality for both localized and metastatic disease and multiple ongoing trials (Table 2) are now examining ways in which to achieve superior clinical outcomes through novel combinations of SBRT with systemic therapy.

**Table 2:** Ongoing SBRT trials combined with systemic therapy.

**Title**	**ClinicalTrial.gov Identifier**	**Intervention/Treatment**	**Phase**
A multi-center trial of androgen suppression with abiraterone acetate, leuprolide, PARP inhibition and stereotactic body radiotherapy in prostate cancer. (ASCLEPluS)	NCT04194554	Non-Randomized Niraparib, Abiraterone, ADT and SBRT	Phase 1 Phase 2
National Danish Protocol. Surgery + SBRT for M1 Prostate Cancer Patients	NCT04086290	Non-Randomized Radical prostatectomy + extended pelvic lymph node dissection in combination with ADT and SBRT.	Phase 1 Phase 2
Management of Oligoprogressive Castration Resistant Prostate Cancer (PCS X)	NCT04070209	Non-Randomized Darolutamide (BAY1841788) + SBRT	Phase 2
Radium-223 and SABR vs. SABR for Oligometastatic Prostate Cancers. (RAVENS)	NCT04037358	Randomized SABR +/- Radium-223	Phase 2
Single dose radiotherapy (SDRT) with or without adjuvant systemic therapy for oligometastatic prostate cancer. (PRELUDE)	NCT04031378	Randomized SBRT +/- systemic therapy for 6 months stratified by disease status (mHSPC vs. mCRPC)	Phase 2
Radioablation with or without androgen depravation therapy in metachronous prostate cancer oligometastasis (RADIOSA)	NCT03940235	Randomized SBRT +/- ADT	Phase 2
Antiandrogen therapy and SBRT in treating patients with recurrent metastatic prostate cancer.	NCT03902951	Non-Randomized Abiraterone Acetate, Apalutamide and ADT + SBRT	Phase 2
Platform study for prostate researching translational endpoints correlated to response to inform use of novel combinations. (PORTER)	NCT03835533	Non-Randomized Nivolumab in combination with: Drug: NKTR-214 (Cohort A) Radiation: SBRT (Cohort B) Drug: CDX-301 (Cohort B and C) Drug: Poly-ICLC (Cohort B) Drug: INO-5151 (Cohort C) Cellectra 2000–Electroporation device (Cohort C)	Phase 1
Prostate cancer with oligometastatic relapse: combining streotactic ablative radiotherapy and durvalumab (MEDI4736). (POSTCARD)	NCT03795207	Randomized SBRT +/- Durvalumab	Phase 2
Local ablative therapy for hormone sensitive oligometastatic prostate cancer. (PLATON)	NCT03784755	Randomized Standard of Care +/- SBRT	Phase 3
Targeted radiotherapy in androgen suppressed prostate cancer patients. (TRAP)	NCT03644303	Non-Randomized SBRT + ADT	
PEACE V: Salvage treatment of oligorecurrent nodal prostate cancer metastases. (STORM)	NCT03569241	Randomized MDT (salvage lymph node dissection or SBRT) + ADT +/- Whole pelvic radiotherapy	Phase 2
Focal radiation for oligometastatic castration resistant prostate cancer. (FORCE)	NCT03556904	Randomized SOC with either abiraterone, enzalutamide or docetaxel +/- Radiation therapy (Either conventional RT or SBRT)	Phase 2
Apalutamide with or without stereotactic body radiation therapy in treating participants with castration resistant prostate cancer. (PILLAR)	NCT03503344	Randomized: Apalutamide +/- SBRT	Phase 2
Stereotactic Body Radiation Therapy in Treating Patients with Localized Prostate Cancer that Have Undergone Surgery	NCT03541850	Non-Randomized Radiations: SBRT Drug: ADT	
Phase II Randomized trial of radiation therapy in oligometastatic mCRPC (ARTO)	NCT03449719	Randomized ADT + Abiraterone/Pred +/- SBRT	Phase 2
Radium Ra 223 dichloride, hormone therapy and stereotactic body radiation therapy in treating patients with metastatic prostate cancer.	NCT03361735	Non-randomized ADT + Radium 223 and SBRT	Phase 2
Radium-223 and radiotherapy in hormone naïve men with oligometastatic prostate cancer to bone. (RROPE)	NCT03304418	Non-Randomized Radium 223 + SBRT	Phase 2
Systemic and tumor directed therapy for oligometastatic prostate cancer.	NCT03298087	Non-Randomized Radical prostatectomy followed by MDT with SBRT + ADT, Abiraterone/Prednisone and Apalutamide	Phase 2
Trial of ADT and SBRT vs. SBRT for intermediate prostate cancer	NCT03056638	Randomized SBRT +/- ADT	Phase 3
A study of definitive therapy to treat prostate cancer after prostatectomy.	NCT03043807	Non-Randomized ADT + Docetaxel followed by adjuvant RT and SBRT to oligometastatic lesions along with Abiraterone and Apalutamide	Phase 2
Pembrolizumab in combination with intratumoral SD-101 therapy.	NCT03007732	Randomized ADT, Abi/Pred, Pembrolizumab and SBRT +/- SD-101	Phase 2
Management of castration resistant prostate cancer with oligometastases. (PCS IX)	NCT02685397	Randomized ADT + Enzalutamide +/- SBRT	Phase 2 Phase 3
Stereotactic body radiation therapy in treating patients with localized high-risk prostate cancer.	NCT02296229	Non-Randomized Radiation: SBRT Drug: Androgen deprivation therapy.	
Fairly brief androgen suppression and stereotactic radiotherapy for high risk prostate cancer—Protocol 2. (FASTR-2)	NCT02229734	Non-Randomized ADT + SBRT	Phase 2
Radiation and androgen ablation for prostate cancer.	NCT01517451	Non-Randomized SBRT + ADT	Phase 1 Phase 2

### Localized Prostate Cancer

ADT can synergize with conventional RT by inducing radiosensitivity due to its effect on tumor cell DNA repair inhibition (55). However, the higher radiation doses achieved with SBRT as well as its immune priming effects may obviate the need for ADT. Alternatively, the immunostimulatory effects of ADT may synergize with SBRT. Several trials are testing whether ADT can improve outcomes in men treated with SBRT alone (NCT01517451, NCT03056638, NCT02296229, NCT02229734). Notable among these studies is NTC03056638, a randomized phase 3 trial, in which patients are randomized to SBRT (8 Gy x 5 fractions) plus placebo vs. SBRT plus 6 months of ADT in men with intermediate risk localized disease. The primary endpoint of the study is pathologic complete response on a post-treatment biopsy 24–30 months after treatment. Larger studies with an MFS endpoint will likely be needed to establish SBRT with ADT as a standard of care for unfavorable intermediate and high risk localized prostate cancer.

### Metastatic Prostate Cancer (Hormone-Sensitive and CRPC)

Based on the impressive safety profile of SBRT used in the context of the STAMPEDE trial, several trials are extending these results to test the hypothesis that SBRT can eradicate MRD in oligometastatic hormone sensitive prostate cancer. Notably, RADIOSA (NCT03940235), a randomized phase II trial in patients with oligometastatic prostate cancer with a maximum of 3 lesions, aims to compare progression-free survival between SBRT to all metastatic lesions alone or in combination with 6 months of ADT. The PLATON trial (NCT03784755) will be a randomized phase 3 trial which will test if SBRT adds to standard of care (SOC) systemic therapy. Patients with newly diagnosed metastatic prostate cancer with up to 3 metastatic lesions amenable to ablative therapy (SBRT or surgery) will be treated with SOC systemic therapy plus local ablative therapy to all sites of disease vs. SOC alone. Of note, radiation to the primary if previously untreated is allowed in both arms as per STAMPEDE. Primary outcome will be failure free survival and secondary outcomes include rPFS, and OS.

Checkpoint inhibitors have had limited benefit in the treatment of patients with metastatic prostate cancer (56). The phase 3 trial of ipilimumab vs. placebo after radiotherapy (8 Gy x 1) in patients with mCRPC that had progressed after docetaxel chemotherapy failed to demonstrate improvements in OS (57). However, the trial reported signs of clinical activity with some patients showing reductions in PSA and improvement in PFS. Later, another randomized phase 3 trial compared ipilimumab vs. placebo in asymptomatic or minimally symptomatic patients with mCRPC naïve to chemotherapy (58). As with the previous trial, ipilimumab did not improve OS but again demonstrated increased PFS and PSA response rates in a subset of the patients. PD-1 blockade has had similarly disappointing results. Nivolumab failed to induce any anti-tumor responses in the first 17 patients with mCRPC treated. In a non-randomized parallel arm study of 258 patients, pembrolizumab treatment was associated with a 6% PSA response rate and 5% soft tissue tumor response rate. Whether SBRT, through its reported immunostimulatory effects, can synergize with anti-PD-(L)-1 checkpoint blockade is the subject of multiple ongoing trials.

Notable among the ongoing trials is POSTCARD, a randomized phase II trial that will test SBRT with or without durvalumab, a monoclonal antibody against PD-L1, in patients with oligometastatic hormone sensitive prostate cancer. Patients with up to 5 bone or lymph node metastases as seen on Ga-68-PSMA PET/CT will be included. Primary outcome will be PFS with secondary outcomes including ADT free survival, time to castrate resistance, prostate cancer specific survival, and OS. This innovative trial will provide an important dataset to assess whether PD-L1 blockade immunotherapy enhances the treatment response to SBRT.

Several trials including the FORCE trial (NCT03556904) are testing whether the addition of radiotherapy to SOC systemic therapy improves objective PFS compared to systemic therapy alone in patients with oligometastatic CRPC. In this trial, patients with up to 5 metastatic lesions will be randomized into the different treatment arms. Systemic therapy will consist of abiraterone, enzalutamide, or docetaxel in combination with ADT. Radiation will range between conventional 30 Gy in 10 fractions to SBRT with 50 Gy in 5 fractions. While this trial does not limit radiation to SBRT alone and allows for conventional radiation schedules, it will help determine whether MDT with radiation adds to the efficacy of different systemic therapies. Correlative studies on peripheral blood and on-treatment biopsies should substantially add to our understanding of the effects of various RT schedules on the TME.

The development of mCRPC is associated with multiple changes to the immune TME. A recent report showed that CRPC cells are more resistant to RT combined with ADT and demonstrate higher levels of MDSCs when compared to hormone sensitive cells (59). These immunosuppressive cells exert a regulatory effect on T cell effector cells promoting an immune permissive environment which makes a T cell response less likely. Multiple trials (NCT03835533, NCT03007732) are testing novel immunotherapeutic combinations along with SBRT with aims to bypass the immunosuppressive microenvironment of CRPC and thus stimulate T cell anti-tumor responses. Notable in this group is PORTER (NCT03835533) cohort B, an active trial testing SBRT (30−50 Gy in 1-5 fractions) in combination with Nivolumab, CDX-301 (a recombinant human Flt3L) (60) and Polyinosinic-polycytidylic acid-poly-l-lysine carboxymethylcellulose (poly-ICLC). CDX-301 has been previously shown in clinical studies to expand key subsets of myeloid and plasmacytoid dendritic cells (60) Poly-ICLC is a synthetic double stranded RNA complex that has been shown to activate toll-like receptor 3 and stimulate intratumoral immune responses in patients (61). By combining radiation with checkpoint inhibition and other novel immunotherapy agents, this and other trials aim to overcome the immunosuppressive environment of CRPC and succeed where previous checkpoint monotherapy trials had failed.

## Conclusion

SBRT is an increasingly used treatment for men with early stage favorable risk prostate cancer. Its use in high risk and metastatic prostate cancer has demonstrated impressive preliminary data with respect to tolerability and oncologic outcomes. Here we have reviewed, the unique radiobiology of SBRT with respect to DNA repair, immunogenic cell death, and downstream T cell priming and the various novel clinical trials that have used SBRT in innovative ways with standard and investigational systemic treatments for prostate cancer. Analysis of specimens derived from these carefully designed studies will likely be instructive as to the biological consequences of SBRT and future opportunities for synergy with systemic treatments.

## Author Contributions

All authors listed have made a substantial, direct and intellectual contribution to the work, and approved it for publication.

## Conflict of Interest

The authors declare that the research was conducted in the absence of any commercial or financial relationships that could be construed as a potential conflict of interest.

## References

[1] Mattiuzzi C Lippi G. Current cancer epidemiology. J Epidemiol Glob Health. (2019) 9:217–22. 10.2991/jegh.k.191008.001 31854162 PMC7310786

[2] National Cancer Institute SEER Cancer Stat Facts: Prostate Cancer. Available online at: https://seer.cancer.gov/statfacts/html/prost.html.

[3] Bekelman JE Rumble RB Chen RC Pisansky TM Finelli A Feifer A Clinically localized prostate cancer: ASCO clinical practice guideline endorsement of an american urological association/American society for radiation oncology/Society of urologic oncology guideline. J Clin Oncol. (2018) 2018:JCO18.00606. 10.1200/jco.18.00606 30183466

[4] Sanda MG Cadeddu JA Kirkby E Chen RC Crispino T Fontanarosa J Clinically localized prostate cancer: aUA/ASTRO/SUO guideline. Part I: risk stratification, shared decision making, and care options. J Urol. (2018) 199:683–90. 10.1016/j.juro.2017.11.095 29203269

[5] Dearnaley D Syndikus I Mossop H Khoo V Birtle A Bloomfield D. Conventional versus hypofractionated high-dose intensity-modulated radiotherapy for prostate cancer: 5-year outcomes of the randomised, non-inferiority, phase 3 cHHiP trial. Lancet Oncol. (2016) 17:1047–60. 10.1016/S1470-2045(16)30102-4 27339115 PMC4961874

[6] Incrocci L Wortel RC Alemayehu WG Aluwini S Schimmel E Krol S. Hypofractionated versus conventionally fractionated radiotherapy for patients with localised prostate cancer (HYPRO): final efficacy results from a randomised, multicentre, open-label, phase 3 trial. Lancet Oncol. (2016) 17:1061–9. 10.1016/S1470-2045(16)30070-5 27339116

[7] Lee WR Dignam JJ Amin MB Bruner DW Low D Swanson GP. Randomized phase III noninferiority study comparing two radiotherapy fractionation schedules in patients with low-Risk prostate cancer. J Clin Oncol. (2016) 34:2325–32. 10.1200/jco.2016.67.0448 27044935 PMC4981980

[8] Catton CN Lukka H Gu CS Martin JM Supiot S Chung PWM. Randomized trial of a hypofractionated radiation regimen for the treatment of localized prostate cancer. J Clin Oncol. (2017) 35:1884–90. 10.1200/JCO.2016.71.7397 28296582

[9] Datta NR Stutz E Rogers S Bodis S. Conventional versus hypofractionated radiation therapy for localized or locally advanced prostate cancer: a Systematic review and meta-analysis along with therapeutic implications. Int J Radiat Oncol Biol Phys. (2017) 99:573–89. 10.1016/j.ijrobp.2017.07.021 29280452

[10] Bruner DW Pugh SL Lee WR Hall WA Dignam JJ Low D. Quality of life in patients with low-Risk prostate cancer treated with hypofractionated vs. conventional radiotherapy: a Phase 3 randomized clinical trial. JAMA Oncol. (2019) 5:664–70. 10.1001/jamaoncol.2018.6752 30763425 PMC6459051

[11] Morgan SC Hoffman K Loblaw DA Buyyounouski MK Patton C Barocas D. Hypofractionated radiation therapy for localized prostate cancer: executive summary of an aSTRO, aSCO, and aUA evidence-Based guideline. Pract Radiat Oncol. (2018) 8:354–60. 10.1016/j.prro.2018.08.002 30322661

[12] Zemplenyi AT Kalo Z Kovacs G Farkas R Beothe T Banyai D. Cost-effectiveness analysis of intensity-modulated radiation therapy with normal and hypofractionated schemes for the treatment of localised prostate cancer. Eur J Cancer Care (Engl). (2018) 27:12430. 10.1111/ecc.12430 26782759

[13] Brenner DJ Hall EJ. Fractionation and protraction for radiotherapy of prostate carcinoma. Int J Radiat Oncol Biol Phys. (1999) 43:1095–101. 10.1016/S0360-3016(98)00438-6 10192361

[14] Vogelius IR Bentzen SM. Dose response and fractionation sensitivity of prostate cancer after external beam radiation therapy: a meta-analysis of randomized trials. Int J Radiat Oncol Biol Phys. (2018) 100:858–65. 10.1016/j.ijrobp.2017.12.011 29485063

[15] Madsen BL Hsi RA Pham HT Fowler JF Esagui L Corman J. Stereotactic hypofractionated accurate radiotherapy of the prostate (SHARP), 33.5 gy in five fractions for localized disease: first clinical trial results. Int J Radiat Oncol Biol Phys. (2007) 67:1099–105. 10.1016/j.ijrobp.2006.10.050 17336216

[16] Jackson WC Silva J Hartman HE Dess RT Kishan AU Beeler WH. Stereotactic body radiation therapy for localized prostate cancer: a Systematic review and meta-Analysis of over 6,000 patients treated on prospective studies. Int J Radiat Oncol Biol Phys. (2019) 104:778–89. 10.1016/j.ijrobp.2019.06.1912 30959121 PMC6770993

[17] Widmark A Gunnlaugsson A Beckman L Thellenberg-Karlsson C Hoyer M Lagerlund M. Ultra-hypofractionated versus conventionally fractionated radiotherapy for prostate cancer: 5-year outcomes of the hYPO-RT-PC randomised, non-inferiority, phase 3 trial. Lancet. (2019) 394:385–95. 10.1016/S0140-6736(19)31131-6 31227373

[18] Brand DH Tree AC Ostler P van der Voet H Loblaw A Chu W. Intensity-modulated fractionated radiotherapy versus stereotactic body radiotherapy for prostate cancer (PACE-B): acute toxicity findings from an international, randomised, open-label, phase 3, non-inferiority trial. The Lancet Oncology. (2019) 20:1531–43. 10.1016/S1470-2045(19)30569-8 31540791 PMC6838670

[19] Morgan SC Hoffman K Loblaw DA Buyyounouski MK Patton C Barocas D. Hypofractionated radiation therapy for localized prostate cancer: executive summary of an aSTRO, aSCO and aUA evidence-Based guideline. J Urol. (2019) 201:528–34. 10.1097/JU.0000000000000071 30759696

[20] Quon HC Ong A Cheung P Chu W Chung HT Vesprini D. Once-weekly versus every-other-day stereotactic body radiotherapy in patients with prostate cancer (PATRIOT): a phase 2 randomized trial. Radiother Oncol. (2018) 127:206–12. 10.1016/j.radonc.2018.02.029 29551231

[21] Malouff TD Stross WC Seneviratne DS Waddle MR May BC Buskirk SJ. Current use of stereotactic body radiation therapy for low and intermediate risk prostate cancer: a National cancer database analysis. Prostate Cancer Prostatic Dis. (2020) 23:349–55. 10.1038/s41391-019-0191-9. 31780782

[22] Gonzalez-Motta A Roach M III. Stereotactic body radiation therapy (SBRT) for high-risk prostate cancer: where are we now? Pract Radiat Oncol. (2018) 8:185–202. 10.1016/j.prro.2017.11.008 29339046

[23] Sydes MR Spears MR Mason MD Clarke NW Dearnaley DP de Bono JS Adding abiraterone or docetaxel to long-term hormone therapy for prostate cancer: directly randomised data from the sTAMPEDE multi-arm, multi-stage platform protocol. Ann Oncol. (2018) 29:1235–48. 10.1093/annonc/mdy072 29529169 PMC5961425

[24] Chi KN Agarwal N Bjartell A Chung BH Pereira de Santana Gomes AJ Given R. Apalutamide for metastatic, castration-Sensitive prostate cancer. N Engl J Med. (2019) 381:13–24. 10.1056/NEJMoa1903307 31150574

[25] Davis ID Martin AJ Stockler MR Begbie S Chi KN Chowdhury S. Enzalutamide with standard first-Line therapy in metastatic prostate cancer. N Engl J Med. (2019) 381:121–31. 10.1056/NEJMoa1903835 31157964

[26] Magee DE Singal RK. Androgen deprivation therapy: indications, methods of utilization, side effects and their management. Can J Urol. (2020) 27:11–6. 32101695

[27] Izard JP Siemens DR. Androgen deprivation therapy and mental health: impact on depression and cognition. European urology focus. (2020). 10.1016/j.euf.2019.11.010. [Epub ahead of print]. 31911085

[28] Parker CC James ND Brawley CD Clarke NW Hoyle AP Ali A. Radiotherapy to the primary tumour for newly diagnosed, metastatic prostate cancer (STAMPEDE): a randomised controlled phase 3 trial. Lancet. (2018) 392:2353–66. 10.1016/S0140-6736(18)32486-3 30355464 PMC6269599

[29] Sweeney CJ Chen YH Carducci M Liu G Jarrard DF Eisenberger M. Chemohormonal therapy in metastatic hormone-Sensitive prostate cancer. N Engl J Med. (2015) 373:737–46. 10.1056/NEJMoa1503747 26244877 PMC4562797

[30] Hellman S Weichselbaum RR. Oligometastases. J Clin Oncol. (1995) 13:8–10. 10.1200/JCO.1995.13.1.8 7799047

[31] Palma DA Olson R Harrow S Gaede S Louie AV Haasbeek C. Stereotactic ablative radiotherapy versus standard of care palliative treatment in patients with oligometastatic cancers (SABR-COMET): a randomised, phase 2, open-label trial. Lancet. (2019) 393:2051–8. 10.1016/S0140-6736(18)32487-5 30982687

[32] Ost P Reynders D Decaestecker K Fonteyne V Lumen N De Bruycker A Surveillance or metastasis-Directed therapy for oligometastatic prostate cancer recurrence: a Prospective, randomized, multicenter phase iI trial. J Clin Oncol. (2018) 36:446–53. 10.1200/JCO.2017.75.4853 29240541

[33] Ost P Reynders D Decaestecker K Fonteyne V Lumen N Bruycker AD Surveillance or metastasis-directed therapy for oligometastatic prostate cancer recurrence (STOMP): five-year results of a randomized phase II trial. J Clin Oncol. (2020) 38:10–. 10.1200/JCO.2020.38.6_suppl.10

[34] Siva S Bressel M Murphy DG Shaw M Chander S Violet J. Stereotactic abative body radiotherapy (SABR) for oligometastatic prostate cancer: a Prospective clinical trial. Eur Urol. (2018) 74:455–62. 10.1016/j.eururo.2018.06.004 30227924

[35] Phillips R Shi WY Deek M Radwan N Lim SJ Antonarakis ES. Outcomes of observation vs. stereotactic ablative radiation for oligometastatic prostate cancer: the oRIOLE phase 2 randomized clinical trial. JAMA Oncology. (2020). 10.1001/jamaoncol.2020.0147. [Epub ahead of print]. PMC7225913 32215577

[36] D'Angelillo RM Francolini G Ingrosso G Ravo V Triggiani L Magli A. Consensus statements on ablative radiotherapy for oligometastatic prostate cancer: a position paper of italian association of radiotherapy and clinical oncology (AIRO). Crit Rev Oncol Hematol. (2019) 138:24–8. 10.1016/j.critrevonc.2019.03.014 31092381

[37] Kalina JL Neilson DS Comber AP Rauw JM Alexander AS Vergidis J. Immune modulation by androgen deprivation and radiation therapy: implications for prostate cancer immunotherapy. Cancers (Basel). (2017) 9:13. 10.3390/cancers9020013 28134800 PMC5332936

[38] Burnette BC Liang H Lee Y Chlewicki L Khodarev NN Weichselbaum RR. The efficacy of radiotherapy relies upon induction of type i Interferon-Dependent innate and adaptive immunity. Cancer Res. (2011) 71:2488–96. 10.1158/0008-5472.CAN-10-2820 21300764 PMC3070872

[39] Matsumura S Wang B Kawashima N Braunstein S Badura M Cameron TO. Radiation-Induced cXCL16 release by breast cancer cells attracts effector t Cells. J Immunol. (2008) 181:3099–107. 10.4049/jimmunol.181.5.3099 18713980 PMC2587101

[40] Dudzinski SO Cameron BD Wang J Rathmell JC Giorgio TD Kirschner AN. Combination immunotherapy and radiotherapy causes an abscopal treatment response in a mouse model of castration resistant prostate cancer. J Immunother Cancer. (2019) 7:218. 10.1186/s40425-019-0704-z 31412954 PMC6694548

[41] Arnold KM Flynn NJ Raben A Romak L Yu Y Dicker AP. The impact of radiation on the tumor microenvironment: effect of dose and fractionation schedules. Cancer Growth Metastasis. (2018) 11:1179064418761639. 10.1177/1179064418761639 29551910 PMC5846913

[42] Lee Y Auh SL Wang Y Burnette B Wang Y Meng Y. Therapeutic effects of ablative radiation on local tumor require cD8+ t cells: changing strategies for cancer treatment. Blood. (2009) 114:589–95. 10.1182/blood-2009-02-206870 19349616 PMC2713472

[43] Lugade AA Moran JP Gerber SA Rose RC Frelinger JG Lord EM. Local radiation therapy of b16 melanoma tumors increases the generation of tumor antigen-specific effector cells that traffic to the tumor. J Immunol. (2005) 174:7516–23. 10.4049/jimmunol.174.12.7516 15944250

[44] Dewan MZ Galloway AE Kawashima N Dewyngaert JK Babb JS Formenti SC. Fractionated but not single-dose radiotherapy induces an immune-mediated abscopal effect when combined with anti-CTLA-4 antibody. Clin Cancer Res. (2009) 15:5379–88. 10.1158/1078-0432.CCR-09-0265 19706802 PMC2746048

[45] Filatenkov A Baker J Mueller AM Kenkel J Ahn GO Dutt S. Ablative tumor radiation can change the tumor immune cell microenvironment to induce durable complete remissions. Clin Cancer Res. (2015) 21:3727–39. 10.1158/1078-0432.CCR-14-2824 25869387 PMC4537844

[46] Dovedi SJ Cheadle EJ Popple AL Poon E Morrow M Stewart R. Fractionated radiation therapy stimulates antitumor immunity mediated by both resident and infiltrating polyclonal t-cell populations when combined with pD-1 blockade. Clin Cancer Res. (2017) 23:5514–26. 10.1158/1078-0432.CCR-16-1673 28533222

[47] Apetoh L Ghiringhelli F Tesniere A Obeid M Ortiz C Criollo A. Toll-like receptor 4-dependent contribution of the immune system to anticancer chemotherapy and radiotherapy. Nat Med. (2007) 13:1050–9. 10.1038/nm1622 17704786

[48] Gupta A Probst HC Vuong V Landshammer A Muth S Yagita H. Radiotherapy promotes tumor-specific effector cD8+ t cells via dendritic cell activation. J Immunol. (2012) 189:558–66. 10.4049/jimmunol.1200563 22685313

[49] Lan J Li R Yin LM Deng L Gui J Chen BQ. Targeting myeloid-derived suppressor cells and programmed death ligand 1 confers therapeutic advantage of ablative hypofractionated radiation therapy compared with conventional fractionated radiation therapy. Int J Radiat Oncol Biol Phys. (2018) 101:74–87. 10.1016/j.ijrobp.2018.01.071 29619980

[50] Morisada M Clavijo PE Moore E Sun L Chamberlin M Van Waes C. PD-1 blockade reverses adaptive immune resistance induced by high-dose hypofractionated but not low-dose daily fractionated radiation. Oncoimmunology. (2018) 7:e1395996. 10.1080/2162402X.2017.1395996 29399393 PMC5790397

[51] Rodriguez-Barbeito P Diaz-Botana P Gago-Arias A Feijoo M Neira S Guiu-Souto J. A model of indirect cell death caused by tumor vascular damage after high-Dose radiotherapy. Cancer Res. (2019) 79:6044–53. 10.1158/0008-5472.CAN-19-0181 31641030

[52] Luke JJ Lemons JM Karrison TG Pitroda SP Melotek JM Zha Y. Safety and clinical activity of pembrolizumab and multisite stereotactic body radiotherapy in patients with advanced solid tumors. J Clin Oncol. (2018) 36:1611–8. 10.1200/JCO.2017.76.2229 29437535 PMC5978468

[53] Formenti SC Rudqvist NP Golden E Cooper B Wennerberg E Lhuillier C. Radiotherapy induces responses of lung cancer to cTLA-4 blockade. Nat Med. (2018) 24:1845–51. 10.1038/s41591-018-0232-2 30397353 PMC6286242

[54] Demaria S Coleman CN Formenti SC. Radiotherapy: changing the game in immunotherapy. Trends Cancer. (2016) 2:286–94. 10.1016/j.trecan.2016.05.002 27774519 PMC5070800

[55] Polkinghorn WR Parker JS Lee MX Kass EM Spratt DE Iaquinta PJ. Androgen receptor signaling regulates dNA repair in prostate cancers. Cancer Discov. (2013) 3:1245–53. 10.1158/2159-8290.CD-13-0172 24027196 PMC3888815

[56] Venturini NJ Drake CG. Immunotherapy for prostate cancer. Cold Spring Harb Perspect Med. (2019) 9:5. 10.1101/cshperspect.a030627 30201787 PMC6496329

[57] Kwon ED Drake CG Scher HI Fizazi K Bossi A van den Eertwegh AJ. Ipilimumab versus placebo after radiotherapy in patients with metastatic castration-resistant prostate cancer that had progressed after docetaxel chemotherapy (CA184-043): a multicentre, randomised, double-blind, phase 3 trial. Lancet Oncol. (2014) 15:700–12. 10.1016/S1470-2045(14)70189-5 24831977 PMC4418935

[58] Beer TM Kwon ED Drake CG Fizazi K Logothetis C Gravis G Randomized, double-Blind, phase iII trial of ipilimumab versus placebo in asymptomatic or minimally symptomatic patients with metastatic chemotherapy-Naive castration-Resistant prostate cancer. J Clin Oncol. (2017) 35:40–7. 10.1200/JCO.2016.69.1584 28034081

[59] Wu CT Chen WC Chen MF. The response of prostate cancer to androgen deprivation and irradiation due to immune modulation. Cancers (Basel). (2018) 11:1. 10.3390/cancers11010020 30587810 PMC6356767

[60] Anandasabapathy N Breton G Hurley A Caskey M Trumpfheller C Sarma P. Efficacy and safety of cDX-301, recombinant human flt3L, at expanding dendritic cells and hematopoietic stem cells in healthy human volunteers. Bone Marrow Transplant. (2015) 50:924–30. 10.1038/bmt.2015.74 25915810 PMC4532305

[61] Kyi C Roudko V Sabado R Saenger Y Loging W Mandeli J. Therapeutic immune modulation against solid cancers with intratumoral poly-ICLC: a Pilot trial. Clin Cancer Res. (2018) 24:4937–48. 10.1158/1078-0432.CCR-17-1866 29950349 PMC6191332

